# Genetic diagnosis and molecular characterization of three novel variations in the phenylalanine hydroxylase gene from Chinese patients with phenylketonuria

**DOI:** 10.17179/excli2026-9271

**Published:** 2026-04-10

**Authors:** Fan Yang, Hua-Feng Li, Wei-Jia Tang, Jin-Ping Zhu, Ji-Gang Qiu, Tian-E. Cai, Li-Mei Yu, Ying Yu

**Affiliations:** 1 Lishui Key Laboratory of Brain Health and Severe Brain Disorders. Lishui Second People’s Hospital, Wenzhou Medical University, Lishui, China; 2 Bio-X Institutes, Key Laboratory for the Genetics of Developmental and Neuropsychiatric Disorders, Ministry of Education, Shanghai Jiao Tong University, Shanghai, China; 3 Department of Medical Genetics, Women & Children's Health Care Hospital of Linyi, Linyi, China; 4 The Research Center for Lin He Academician New Medicine, Institutes for Shanghai Pudong Decoding Life, Shanghai, China; 5 Medical Genetics & Antenatal Diagnosis Center, Hainan Branch, Shanghai Children's Medical Center, School of Medicine, Shanghai Jiao Tong University, Sanya, China; 6 Key Laboratory of Cell Engineering in Guizhou Province, Affiliated Hospital of Zunyi Medical University, Zunyi, China; 7 Key Laboratory of Molecular Medicine for Women and Children of Hainan Province, Hainan Branch, Shanghai Children’s Medical Center, School of Medicine, Shanghai Jiao Tong University, Sanya, 572000, China

**Keywords:** phenylketonuria, hyperphenylalaninemia, phenylalanine hydroxylase, novel deletion, pathogenic variation, mRNA and protein expression, enzyme activity

## Abstract

Loss-of-function variants in the human *phenylalanine hydroxylase* (*PAH*) gene are the most common genetic causal factors for Phenylketonuria (PKU). Currently, a broad spectrum of variations is recognized in the human *PAH* gene. However, the molecular function and clinical significance of some novel *PAH* variants remain unclear. Here, we report on five PKU-affected families carrying three novel *PAH* variants, including one missense variant (*PAH*: c.271C>A (p.Leu91Met)) and two deletions (*PAH*: c.206_208delCTT (p.Ser70del) and *PAH*: c.541_544delGAGG (p.Glu181Lysfs*13)). These variations constitute different compound heterozygous genotypes with other known pathogenic variants such as* PAH*: c.721C>T (p.Arg241Cys), *PAH*: c.168+5G>C, and *PAH*: c.1238G>C (p.Arg413Pro), which probably led to the patients' PKU etiopathology. qRT-PCR and immunoblotting showed that the protein levels of PAH (S70del) and PAH (E181Kfs*13) were significantly reduced compared with the wild-type control, although their transcript levels were not. Also, the enzyme activity of PAH (S70del) and PAH (E181Kfs*13) mutants was significantly decreased relative to the wild type (*P* < 0.001). *PAH*: c.271C>A (p.Leu91Met) had no significant effect on *PAH* mRNA and protein levels or enzyme activity. Collectively, our data demonstrate that the two deletions *PAH*: c.206_208delCTT and *PAH*: c.541_544delGAGG are clinically significant for pathogenicity. Our findings are anticipated to contribute to the advancement of prenatal diagnosis, population-based carrier screening, and genetic counseling for individuals affected by PKU, and is expected to help reduce the incidence of PKU and ameliorate the associated disease burden.

See also the graphical abstract(Fig. 1).

## Abbreviations

BH_4_: tetrahydrobiopterin

HPA: hyperphenylalaninemia

HSP: heat shock protein

PAH: phenylalanine hydroxylase

PCR: polymerase chain reaction

Phe: phenylalanine

PKU: phenylketonuria

Tyr: tyrosine

## Introduction

Phenylketonuria (PKU, OMIM 261600) is one of the most common human autosomal recessive monogenetic diseases. It is characterized by phenylalanine (Phe) metabolism deficiency and elevated Phe plasma levels, which is primarily caused by loss-of-function variants in the *phenylalanine hydroxylase* (*PAH*) gene, but also partially by defects in the metabolism of the co-factor tetrahydrobiopterin (BH_4_), or by deficiency of heat shock protein (Hsp) family members like Hsp40 DnaJC12 (Anikster et al., 2017[3], Bartholome, 1974[4]). The *PAH* gene encodes the phenylalanine-4-hydroxylase enzyme, which catalyzes the rate-limiting step of the L-phenylalanine (L-Phe) to L-tyrosine (L-Tyr) hydroxylation. Clinically, PKU is a severe form of hyperphenylalaninemia (HPA), defined by blood Phe concentrations exceeding 120 μmol/L (i.e., 2 mg/dl) and a Phe/Tyr ratio > 2.0. Plasma Phe concentrations in patients with milder and classical PKU, respectively, are between 600 and 1200 μmol/L and > 1200 μmol/L (Blau et al., 2010[7], Guthrie and Susi, 1963[23]). Patients with classical severe PKU usually have musty odor, severe intellectual disability, and light pigmentation (Blau et al., 2010[7]). Although alternative approaches such as gene therapy have recently emerged, dietary restriction of Phe is still the most effective treatment of PKU or milder HPA (Lichter-Konecki and Vockley, 2019[35], MacDonald et al., 2020[36]). This classic intervention has been used for more than 50 years, but it has been rarely revised (Bickel et al., 1953[5], Sarkissian et al., 1999[45]). Epidemiologically, the incidence of HPA shows great regional variability (van Spronsen et al., 2021[55]). Compared to high-incidence regions in Europe, such as Italy, HPA is much less prevalent in Asia. In China, the average incidence of HPA is 1:15,924 live births (Xiang et al., 2019[61]). Currently, more than 1,180 variations in the human *PAH* gene are known, many of which are clinically significant (Hillert et al., 2020[24]). Although novel *PAH* variations are still being revealed, little is known about their clinical significance. Thus, continued in-depth investigation is required to better understand the etiopathology and molecular mechanisms underlying HPA and PKU, improve genetic diagnosis and prevention of the diseases, and develop new therapies.

## Materials and Methods

### Subject recruitment

The protocol of this study was reviewed and approved by the Ethics Committee of Hainan Branch, Shanghai Children's Medical Center, School of Medicine, Shanghai Jiao Tong University, Sanya (Approval No. SYFYIRB2023042), and the Ethics Committee of Women & Children's Health Care Hospital of Linyi (Approval No. 2013-YXLL-001). All patients with PKU or HPA were clinically diagnosed through comprehensive analysis of blood Phe level detection results and clinical feature data. Written informed consent was obtained from all participants including PKU or HPA patients or their legal guardian, and their family members, before enrollment. Besides, written informed consent to publish was acquired from each patient/participant. A total of 110 trio families with PKU or HPA were recruited. Approximately 2 mL peripheral blood was collected from each subject with an EDTA-anticoagulant vacutainer. In addition, amniotic fluid biospecimens were collected from pregnant women according to standard medical operating procedures for genetic analysis of fetal *PAH* gene variations.

### Genetic analysis of PAH gene variations

Genomic DNA (gDNA) was purified from all participants' blood cells using a Genomic DNA Purification Kit (TIANGEN, Cat no. DP304). All thirteen exon sequences of the *PAH* gene were amplified by polymerase chain reaction (PCR), followed by agarose gel electrophoresis and Sanger sequencing. Primers used for amplifying the exons and their bilateral exon-intron boundary sequences are given in Supplementary Table S1. Different types of variations including deletions/insertions, missense variants, nonsense variants, and splicing errors, were further confirmed by bidirectional sequencing. 

### Detection of PAH mRNA and protein expression

Full-length *PAH* coding sequences (1359 bp) were amplified and cloned into pCMV-myc-N plasmids (Yidao Biotech, Nanjing, China). The restriction enzymes EcoR I and Xho I were used for the recombination reaction. The three variations *PAH*: c.271C>A, *PAH*: c.206_208del, and* PAH*: c.541_544del were then generated by site-directed mutagenesis. Primers used for generating the variations are given in Table S1. Successful generation of the target sites without random PCR errors was further confirmed by Sanger sequencing. Four recombinant vectors, expressing wild-type *PAH* and the three mutated forms, were transfected into HEK-293T cells (FuHeng Biotech, Cat. no. FH0821), followed by continued culture with DMEM medium (ThermoFisher, Waltham, USA) for 24 h. Finally, cell cultures were centrifuged, and total RNA was extracted and reverse transcribed into first-strand cDNA. mRNA levels were detected with real-time quantitative PCR (qRT-PCR). Primers used for qRT-PCR are given in Table S1. Total proteins were also extracted from the cells for immunoblotting. An Myc Tag Mouse mAb antibody (Vazyme, Cat. no. RA1005-01) was used for detecting the expression level of recombinant proteins (~53 kDa) that tagged Myc on the N terminal (1:3000 dilution), and a β-Actin antibody (Affinity Biosciences, Cat. no. T0022) (1:160000 dilution) was used for detecting the reference protein β-ACTIN (~42 kDa). The expression levels of PAH and ACTIN proteins were quantitatively analyzed using ImageJ software (bundled with 64-bit Java 8 , National Institutes of Health, USA). The relative expression levels of PAH in each group were represented as the ratio of PAH vs ACTIN, and normalization analysis was performed. *PAH* mRNA and protein levels between different groups were compared using one-way ANOVA & Tukey HSD or Kruskal-Wallis & Dunn test, according to the normal distribution of data and the homogeneity of variance.

### Measurement of enzyme activity

Briefly, 100 μg of total protein released from the lysed cells was pipetted into a PAH enzyme activity reaction, which contained 0.5 mM L-phenylalanine, 1.0 mM tetrahydrobiopterin (Macklin, Shanghai, China), and 40 U catalase (Sangon Biotech, Shanghai, China). The reaction mixture was incubated at 37 °C for 60 min. The concentration of PAH-produced L-tyrosine and residual L-phenylalanine was determined with an Amino Acid Analyzer (Hitachi, Tokyo, Japan), using the following formulas: Relative amount of Tyr (%) = Tyr concentration (μM) / (Phe + Tyr) concentration (μM) × 100 %; absolute amount of Tyr (μM) = relative amount of Tyr (%) × initial concentration of Phe (500 μM). Samples from different groups were compared using Kruskal-Wallis & Dunn test.

### Protein sequence alignment and structure modeling

PAH protein sequences of different organisms were downloaded from NCBI (https://www.ncbi.nlm.nih.gov) and aligned with PRALINE (https://www.ibi.vu.nl/programs/pralinewww/) using default parameters. Amino acid conservation at different alignment positions was visualized with PRALINE's color scoring scheme (0 (blue) = least conserved, 10 (red) = most conserved). Structure modeling of the wild-type and the mutated PAH proteins was performed with SWISS-MODEL (https://swissmodel.expasy.org) under default parameters.

## Results

### Identification of three novel PAH variations

**Figure 1:**
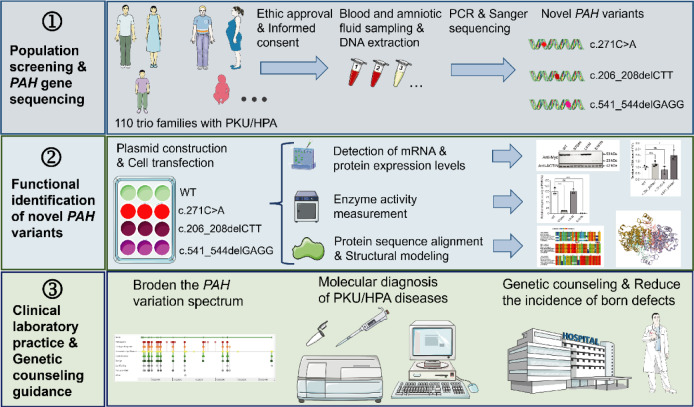
Graphical abstract

To reduce the incidence of PKU/HPA in the region, we launched a long-term population-screening initiative that performs comprehensive *PAH* gene sequencing in affected families, delineates the clinical significance of newly identified variants, and establishes genotype-phenotype causality, with the ultimate goal of preventing PKU/HPA and related birth defects at the local level (see Figure 1(Fig. 1): Graphical abstract). A total of 110 trio families with PKU or HPA were recruited for this study. The demographic and clinical characteristics of the entire cohort are summarized in Supplementary Table S3. Briefly, the cohort comprised 48 (43.6 %) male, 54 (49.1 %) female probands, and 8 (7.3 %) fetus. The mean age at diagnosis was 3.8 ± 3.5 years. Based on plasma Phe levels at diagnosis, 55 patients (50.0 %) were classified as classic PKU (Phe > 1200.0 μmol/L), 33 patients (30.0 %) as mild PKU (Phe 600.0-1200.0 μmol/L), and 14 patients (12.7 %) as HPA (Phe 120.0-600.0 μmol/L). The mean plasma Phe concentration at diagnosis was 1266.2 ± 575.6 μmol/L (range: 156.0-3012.6 μmol/L).

Molecular genetic diagnosis identified three novel *PAH* variations, including two deletion (*PAH*: c.206_208delCTT and *PAH*: c.541_544delGAGG) and one missense variant (*PAH*: c.271C>A), in five PKU probands (Table 1(Tab. 1)). Clinical characteristics and plasma Phe concentrations of the five patients are shown in Table 1(Tab. 1). All five patients presented with classic symptoms of PKU in the childhood or early infancy. The most common clinical manifestations at diagnosis included poor feeding (2/5, 40 %), growth and developmental delay (3/5, 60 %), language expression impairment (3/5, 60 %), intellectual impairment (3/5, 60 %), learning disability (3/5, 60 %), musty odor (3/5, 60 %), and lethargy or irritability (5/5, 100 %). Three patients (P1, P2, and P3) presented with hypopigmentation of hair and skin. Patient 3 (P3), who carried the c.541_544delGAGG (p.Glu181Lysfs*13) variant in combination with c.721C>T (p.Arg241Cys), presented with the most severe clinical phenotype, including moderate growth and developmental delay, language disability, anorexia, malnutrition, hyperactivity, irritability, lethargy, attention deficit, moderate intellectual development delay, hypopigmentation of skin and hair, and musty odor. This patient also showed mild developmental delay at follow-up, suggesting a potential correlation between the compound heterozygosity of c.541_544delGAGG/c.721C>T and more severe disease manifestation, although we caution that this observation is based on a single patient and requires validation in larger cohorts.

All patients were immediately initiated on Phe-restricted diets supplemented with Phe-free medical formulas following diagnosis. Regular monitoring of plasma Phe levels and dietary adjustments were performed according to established clinical guidelines. At the time of last follow-up (ranging from 2 to 5 years of age), four patients demonstrated normal developmental milestones with well-controlled plasma Phe levels (120.0-677.4 μmol/L). Patient P3 showed mild developmental and intellectual delay but achieved independent walking and basic language skills.

These detailed clinical data facilitate preliminary genotype-phenotype correlation analysis. Notably, patients (P3, P4, and P5) carrying the c.541_544delGAGG (p.Glu181Lysfs*13) variant tended to present with higher initial plasma Phe levels (1320.0, 3012.6, and 1599.6 μmol/L, respectively) compared to other patients, suggesting that this novel frameshift deletion may be associated with more severe biochemical and clinical phenotypes. However, we emphasize that these observations are preliminary given the limited sample size and require confirmation in future studies with larger patient cohorts.

**Figure 2:**
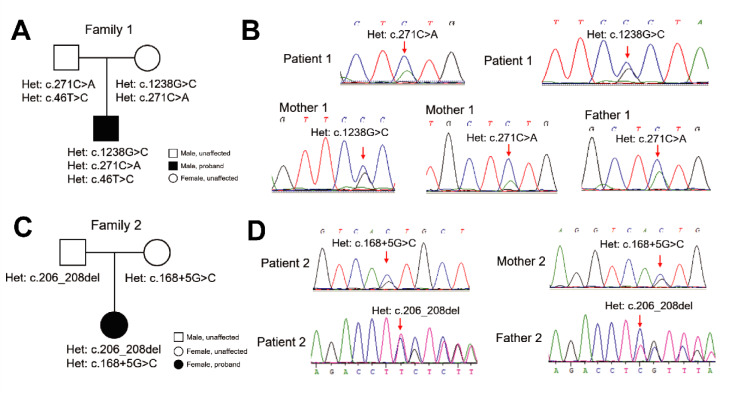
Identification of the novel *PAH* variations c.271C>A and *PAH*: c.206_208delCTT. (A) Pedigree analysis of PKU family 1. (B) Sanger sequencing revealed the heterozygous and novel missense variant *PAH*: c.271C>A in patient 1, mother 1, and father 1, and the heterozygous missense variation* PAH*: c.1238G>C in patient 1 and mother 1. (C) Pedigree analysis of PKU family 2. (D) Sanger sequencing revealed the heterozygous and novel 3-bp deletion *PAH*: c.206_208delCTT in patient 2 and father 2, and the heterozygous splicing variant *PAH*: c.168+5G>C in patient 2 and mother 2.

The heterozygous missense variant *PAH*: c.271C>A (p.Leu91Met) was discovered in patient 1, a 5-year-old boy from family 1. This novel variant constitutes a compound heterozygous genotype with the other two missense variations *PAH*: c.1238G>C (p.Arg413Pro) and *PAH*: c.46T>C (p.Ser16Pro) (Figure 2A(Fig. 2)). Sanger sequencing showed that *PAH*: c.1238G>C was inherited from the mother; unexpectedly, both mother and father carried the novel variant *PAH*: c.271C>A (Figure 2B(Fig. 2)). Other variants with (likely) benign or uncertain significance identified in family 1 are shown in the Supplementary Table S2. Blood Phe concentration of patient 1 was 1320.0 μmol/L and could be reduced to 840.0 μmol/L after dietary restriction treatment (Table 1(Tab. 1)). 

The novel 3-bp deletion *PAH*: c.206_208delCTT was discovered in patient 2, a 4-year-old girl, who also carried the heterozygous variant *PAH*: c.168+5G>C (Figure 2C(Fig. 2)). Sanger sequencing revealed that the 3-bp deletion and the splicing variant were inherited from both father and mother (Figure 2D(Fig. 2)). The blood Phe concentration in patient 2 was 1440.0 μmol/L; it decreased to 677.4 μmol/L after dietary restriction (Table 1(Tab. 1)). Other benign variations identified in family 2 are shown in Supplementary Table S2.

**Figure 3:**
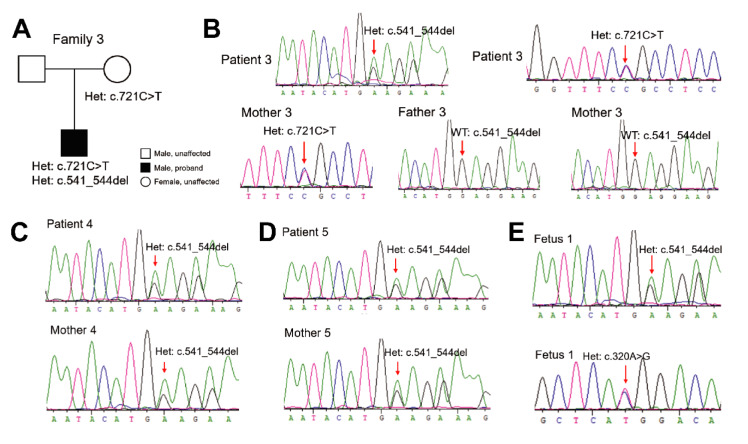
Identification of the novel deletion *PAH*: c. c.541_544delGAGG. (A) Pedigree analysis of PKU family 3. (B) Sanger sequencing revealed the heterozygous 4-bp deletion *PAH*: c.541_544delGAGG in patient 3, and the heterozygous missense variation *PAH*: c.721C>T in patient 3 and mother 3. The 4-bp deletion was absent from samples of father 3 and mother 3. (C) and (D) Sanger sequencing revealed the heterozygous deletion *PAH*: c.541_544delGAGG in patient 4 and mother 4 from PKU family 4, and in patient 5 and mother 5 from PKU family 5. (E) Sanger sequencing revealed the heterozygous deletion *PAH*: c.541_544delGAGG and missense variant *PAH*: c.320A>G in fetus 1. Het: heterozygous, WT: wild type.

In addition, a 4-bp deletion, *PAH*: c.541_544delGAGG, was identified from three PKU patients and one fetus, including patients 3, 4, 5, and fetus 1 from different families. In family 3, a compound heterozygosity genotype of *PAH*: c.541_544delGAGG and *PAH*: c.721C>T (p.Arg241Cys) was revealed in patient 3, a 5-year-old boy (Figure 3A(Fig. 3)). Sanger sequencing showed that the missense variation *PAH*: c.721C>T was inherited from the mother (Figure 3B(Fig. 3)). However, the 4-bp deletion *PAH*: c.541_544delGAGG was absent from the samples of both father and mother of patient 3 (Figure 3B(Fig. 3)), suggesting that it was likely an embryonic *de novo* variation. Blood Phe concentration of patient 3 was 1320.0 μmol/L before treatment and largely reduced to 120.0 μmol/L after excessive dietary restriction (Table 1(Tab. 1)). The same deletion was also found in two other PKU-affected newborns, namely patients 4 and 5 (Figures 3C and D(Fig. 3), Table 1(Tab. 1)), and was in both cases inherited from the mother (Figures 3C and D(Fig. 3)). However, we failed to recruit their fathers for detailed molecular analysis of *PAH* variations. The respective blood Phe concentrations of patients 4 and 5 were 3012.6 μmol/L and 1599.6 μmol/L. After dietary treatment, the concentrations were largely reduced to 600.0 μmol/L and 534.0 μmol/L, respectively (Table 1(Tab. 1)). Interestingly, genetic testing of an amniotic fluid sample from one pregnant subject also detected the deletion *PAH*: c.541_544delGAGG, as well as the missense variation *PAH*: c.320A>G (p.His107Arg) (Figure 3E(Fig. 3), Table 1(Tab. 1)). Unfortunately, we could not analyze their genetic origin because the couple failed to enroll. Other benign variations identified in families 3, 4, and 5 are shown in Supplementary Table S2. Sanger sequencing further confirmed that the three novel variants were absent in > 300 healthy subjects recruited in our study.

### Functional characterization of the novel missense variant PAH: c.271C>A

**Figure 4:**
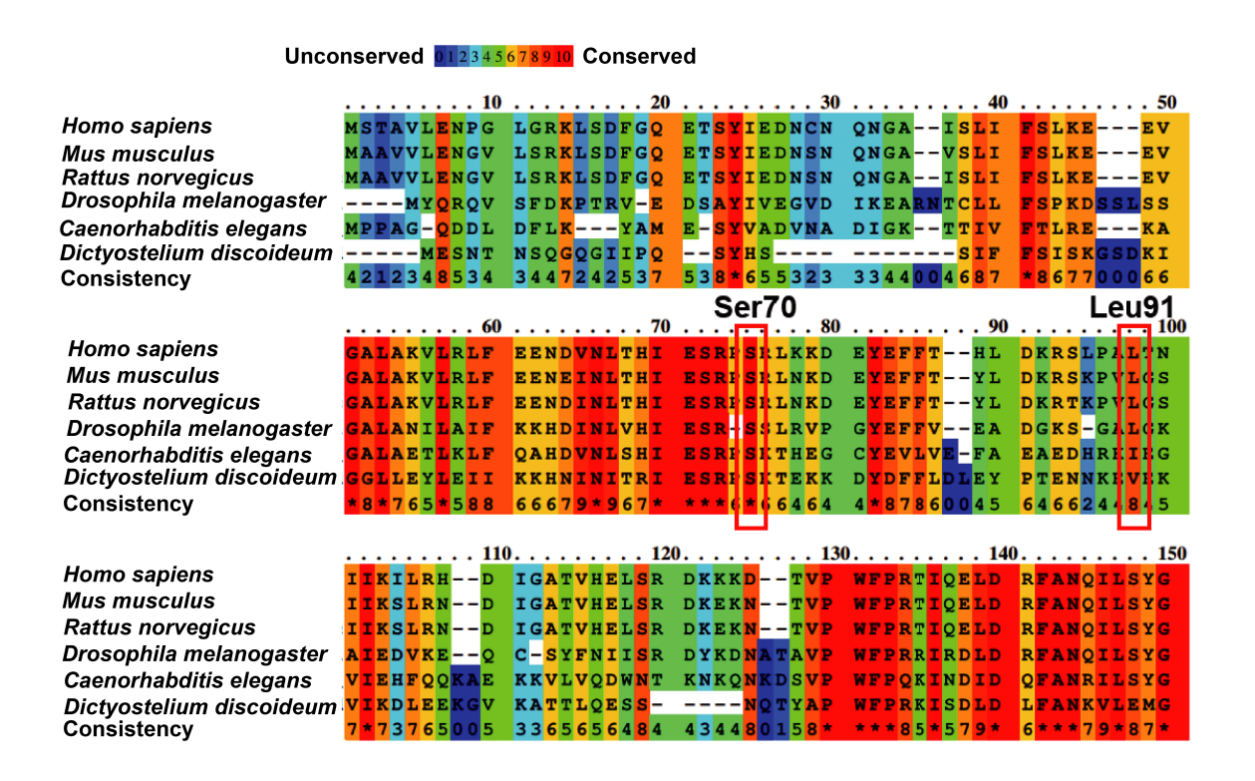
Evolutionary conservation analysis of PAH protein sequences from multiple organisms. Alignment of partial PAH protein sequences (residues 1-139) from *Homo sapiens*, *Mus musculus*, *Rattus norvegicus*, *Drosophila melanogaster*, *Caenorhabditis elegans*, and *Dictyostelium discoideum*. The red box indicates the Ser70 and Leu91 residues. The online tool PRALINE was used to perform the analysis. Values ranging from 0 to 10 represent the conservation degree of each amino acid residue.

Previous studies suggested that the missense variant *PAH*: c.1238G>C (p.Arg413Pro) was clinically pathogenic (Liang et al., 2014[34], Okano et al., 1998[41], 2004[42], 2011[44], Shi et al., 2012[46], Tao et al., 2015[53], Wang et al., 1991[56], Zurfluh et al., 2008[67]). The novel missense variant *PAH*: c.271C>A discovered here results in methionine (Met) replacement of leucine (Leu) at position 91. According to the multiple sequence alignment of PAH proteins from different organisms, the amino acid residue Leu91 is evolutionarily conserved across multiple species such as human, mouse, rat, fruit fly, roundworm, and slime mold (Figure 4(Fig. 4)). Functional prediction analysis using MutationTaster showed that *PAH*: c.271C>A (Leu91Met) was likely a deleterious variant. 

**Figure 5:**
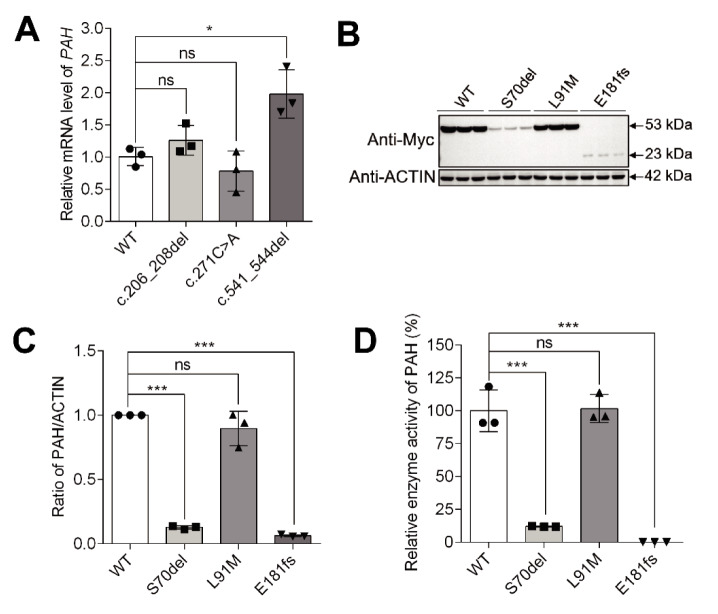
Functional analysis of the three novel *PAH* variations. (A) qRT-PCR results of the transcription level of transiently expressed wild-type* PAH* and the three *PAH *mutants (c.271C>A, c.206_208delCTT, and c.541_544delGAGG) in HEK-293T cells. (B) Immunoblotting result of the abundances of HEK-293T cell-expressed recombinant proteins of wild-type PAH and the three PAH mutants (p.Ser70del, p.Leu91Met, and p.Glu181Lysfs*13) fused with an Myc tag on the N terminal. Anti-Myc (1:3000 dilution) and anti-ACTIN antibody (1:160000 dilution) were used for detection of target and reference proteins. (C) Relative expression levels of wild type and the three mutated PAH were represented as ratio of PAH/ACTIN bands intensity that normalized to wild type control. (D) Enzyme activity testing results of HEK-293T cell-expressed proteins of wild-type PAH and the three PAH mutants. Bar plots in (A), (C), and (D) represent mean ± standard deviation (SD) of three biological replicates. Samples were compared using one-way ANOVA & Tukey HSD test or Kruskal-Wallis & Dunn test. ns: not significant. *: *P* < 0.05, **: *P* < 0.01, ***: *P* < 0.001.

**Figure 6:**
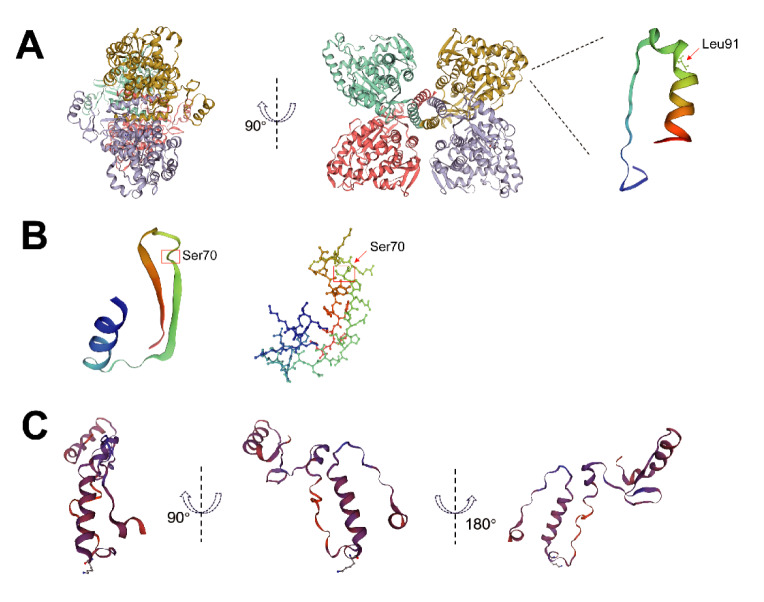
Structural analysis of the three mutated PAH proteins. (A) Structural modeling of the mutated PAH (p.Leu91Met) tetramer. Left panel: The PAH (p.Leu91Met) tetramer represented by 90°-leftward rotation. Middle panel: structure of the tetramer represented in cartoon form; the four monomers are denoted with different colors. Right panel: structure of the polypeptide ranging from amino acid residue 71 to 102 of the PAH regulatory domain (RD). Red arrow indicates position of Leu91 on the third helix (residue 91 to 102). Sticks indicate the Leu91 residue. (B) The position of the Ser70 residue on the beta strand and helix structure ranging from amino acid residue 47 to 83 of PAH RD. Ser70 is indicated by the red box and arrow. Left panel: cartoon form. Right panel: ball & stick form. (C) Structural modeling of the mutated PAH (p.Glu181Lysfs*13) protein. Middle panel: structure of the truncated PAH (p. Glu181Lysfs*13) protein represented in cartoon form. Left and right panel: structure of PAH (p. Glu181Lysfs*13) represented by 90°-leftward rotation and 180°-rightward rotation, respectively. The online tool SWISS-MODEL was used for the analysis.

To determine its effect on *PAH* mRNA and protein expression, as well as PAH enzyme activity, we generated it by site-directed mutagenesis and transfected the mutated *PAH* (c.271C>A)-expressing vector into HEK-293T cells. qRT-PCR and immunoblotting results showed that the transcript and protein expression levels of the mutant *PAH* (c.271C>A) were comparable to those of wild-type *PAH* (Figures 5A-C(Fig. 5)). Moreover, the homozygous c.271C>A variant barely affected the enzymatic activity of PAH (Figure 5D(Fig. 5)). The substitution of Leu91 with Met occurred in the third helix of the regulatory domain (Figure 6A(Fig. 6)), which might affect the regulation potential of the PAH mutant. According to these *in vitro* data, the variant *PAH*: c.271C>A seems to be benign. However, we cannot completely rule out the possibility that the classical PKU phenotype of patient 1 was due to the loss-of-function variation c.1238G>C (p.Arg413Pro) that compounds with heterozygous c.271C>A (Leu91Met) in the *PAH* gene.

### Deletion PAH: c.206_208delCTT is pathogenic

It is well known that *PAH*: c.168+5G>C is a pathogenic variation (Alibakhshi et al., 2014[2], Buratti et al., 2007[9], Georgiou et al., 2012[20], Jeannesson-Thivisol et al., 2015[26], Kostandyan et al., 2011[29], Murad et al., 2013[40], Sterl et al., 2013[50], Zare-Karizi et al., 2011[62], Zygulska et al., 1993[68]). c.206_208delCTT led to a non-frameshift deletion of the Ser70 residue, which is evolutionarily highly conserved across multiple species (Figure 4(Fig. 4)). qRT-PCR and immunoblotting showed that although the transcript expression level of mutated *PAH* (c.206_208del) was comparable to the wild type group (Figure 5A(Fig. 5)), the abundance of the PAH (Ser70del) protein was significantly reduced relative to wild-type PAH (*P* < 0.001) (Figures 5B and C(Fig. 5)). Besides, the mutated PAH (Ser70del) protein showed significantly decreased enzymatic activity compared to that of wild type PAH (*P* < 0.001) (Figure 5D(Fig. 5)). The Ser70 residue is located in the second beta strand (residues 62-73) of the regulatory domain (Figure 6B(Fig. 6)). The deletion of Ser70 probably disrupts the regulatory activity and catalytic potency of PAH. Taken together, these findings suggest that the deletion *PAH*: c.206_208delCTT (p.Ser70del) was definitely pathogenic, and the compound heterozygous genotype of *PAH*: c.206_208delCTT and* PAH*: c.168+5G>C is the genetic causal factor responsible for PKU pathology of patient 2.

### Deletion PAH: c.541_544delGAGG is pathogenic

Previous studies indicated that *PAH*: c.721C>T (p.Arg241Cys) was a pathogenic variant (Acosta et al., 2001[1], Chien et al., 2004[12], Guldberg et al., 1996[22], Kim et al., 2006[27], Kure et al., 1999[30], Lee et al., 2004[31], 2008[32], Liang et al., 2014[34], Michiels et al., 1998[38], Okano et al., 1994[43], 2004[42], Shintaku et al., 2004[47], Spaapen et al., 2001[49], Takarada et al., 1993[52], Tao et al., 2015[53], Zhu et al., 2010[65], Zurfluh et al., 2008[67]). c.541_544delGAGG resulted in a protein-coding frameshift and a premature stop codon, leading to a truncated PAH (Glu181Lysfs*13) form (192 aa, ~22.19 kDa). qRT-PCR showed that the transcript level of the *PAH *(c.541_544del) mutant was significantly higher than that of wild-type *PAH *(*P* < 0.05) (Figure 5A(Fig. 5)). However, the truncated PAH (Glu181Lysfs*13) protein was clearly less abundant (*P* < 0.001) (Figures 5B and C(Fig. 5)), suggesting that the truncated version of PAH protein was extremely unstable and rapidly degraded. Furthermore, the enzyme activity of PAH (Glu181Lysfs*13) mutant was completely abolished (Figure 5D(Fig. 5)). The partial catalytic domain (residues 121-427) and the whole oligomerization domain (residues 428-452) on the C-terminal were missing from the truncated PAH (Glu181Lysfs*13) protein (Figure 6C(Fig. 6)), which blocks the tetramerization of the monomeric PAH (Glu181Lysfs*13) mutant. Taken together, these results demonstrate that *PAH*: c.541_544delGAGG was a clinically significant variation with severe pathogenicity. Based on the genetic, biochemical, and structural modeling results, the compound heterozygosity condition of *PAH*: c.541_544delGAGG and *PAH*: c.721C>T (p.Arg241Cys) accounted for the molecular etiopathology of PKU in patient 3.

## Discussion

The monomeric PAH protein consists of three functional domains: a regulatory domain (RD, residues 1-120) at the N terminal, a central catalytic domain (CD, 121-427), and a multimerization domain (MD, 428-452) at the C terminal (Erlandsen et al., 1997[16], Fusetti et al., 1998[19], Hufton et al., 1995[25], Kobe et al., 1999[28]). The RD regulates enzyme activity, the CD is involved in iron-mediated incorporation of oxygen into the amino acid substrate and the subsequent hydroxylation reaction, and the MD mediates the tetramerization of the monomeric PAH protein (Fitzpatrick, 2003[18]). Under physiological conditions, PAH proteins are normally found in an equilibrium state between tetrameric, dimeric, and oligomeric forms (Martinez et al., 1995[37]). Activation of PAH requires the binding of Phe to the RD and subsequent conformational changes. Previous studies have shown that some, also naturally occurring, variations in the RD destabilize PAH and decrease its concentration by facilitating aggregation and degradation processes (Bjorgo et al., 1998[6], Eiken et al., 1996[15], Gjetting et al., 2001[21], Waters et al., 1998[59][60], 1999[57], 2000[58]). It has been further demonstrated that some variants in the vicinity of the Leu91 residue have significant effects on PAH stability and enzymatic activity. For instance, the missense variant p.Tyr92Ile reduces enzyme activity of PAH by ~25 % and is responsive to BH_4_ administration (Fiori et al., 2005[17], Leuzzi et al., 2006[33], Mirisola et al., 2001[39]). Further, p.Pro89Ser, in the compound heterozygous genotype with Arg408Gln, can induce a mild HPA phenotype (Chen et al., 2015[11], Su et al., 2019[51]). Finally, p.Ser87Arg, which is responsive to BH_4_ addition (Bueno et al., 2013[8], Desviat et al., 2004[14], Jeannesson-Thivisol et al., 2015[26]), can lead to ~25-82 % reduction of PAH activity (Couce et al., 2013[13]). Patients carrying both p.Ser87Arg and frame-shift variation p.Gly352fs, splicing variant c.1065+1G>A, or missense variant p.Ser349Pro, show mild HPA symptoms (Bueno et al., 2013[8], Desviat et al., 2004[14], Jeannesson-Thivisol et al., 2015[26]).

In this study, we identified a novel missense variant,* PAH*: c.271C>A (p.Leu91Met). We found that the Leu91 residue is evolutionarily conserved across multiple species (Figure 4(Fig. 4)) and that *PAH*: c.271C>A (p.Leu91Met) is likely a deleterious variant. The Met substitution of Leu91 is located in the third helix of the RD (Figure 6A(Fig. 6)) and thus might influence its regulation activity. c.271C>A slightly reduced the transcript and protein level of *PAH* compared to the wild-type control (Figures 5A-C(Fig. 5)). Moreover, the enzyme activity of PAH (Leu91Met) barely differed from wild-type PAH (Figure 5D(Fig. 5)). These results suggest that the missense variant* PAH*: c.271C>A (p.Leu91Met) is benign. However, we cannot rule out that it has mild pathogenic effects. In fact, patients carrying it together with the pathogenic variation c.1238G>C (p.Arg413Pro) showed classical PKU symptoms (Table 1(Tab. 1)). Nonetheless, based on the combined molecular genetics, biochemical, functional, and clinical evidence, we speculate that homozygous c.271C>A (p.Leu91Met) is unable to cause obvious HPA or PKU phenotypes, because the residual enzyme activity of the Leu91Met mutant was sufficient to convert Phe to Tyr. However, p.Leu91Met could still have an effect when the other *PAH* allele harbors severe pathogenic variations such as c.1238G>C or a functional null variant, which might affect the assembly of a biologically functional PAH tetramer and the normal conversion of Phe to Tyr. For example, individuals with the compound heterozygous genotype of *PAH*: c.271C>A plus a pathogenic variation such as c.1238G>C are likely to develop HPA or PKU. Considering the clinical evidence of our patient 1, further functional, structural, and BH_4_-loading studies are required to determine whether p.Leu91Met is a mild HPA and BH_4_-responsive variation. 

The novel 3-bp deletion c.206_208delCTT discovered in this study led to a non-frameshift deletion of the Ser70 residue, which generated the same alteration in protein level as the known pathogenic variation c.208_210delTCT (Cao et al., 2014[10], Hillert et al., 2020[24], Lee et al., 2004[31], Liang et al., 2014[34], Okano et al., 1998[41], 2011[44], Song et al., 2005[48], Su et al., 2019[51], Tao et al., 2015[53], 2021[54], Zhao et al., 2019[63], Zhou et al., 2012[64], Zhu et al., 2013[66]). Immunoblotting and biochemical assays showed that homozygous c.206_208delCTT greatly reduces the PAH protein level and enzyme activity (~12 % less than the wild-type control) (Figures 5B-D(Fig. 5)). Because the Ser70 residue is located in the second beta strand (residues 62-73) of PAH-RD (Figure 6B(Fig. 6)), its absence greatly disrupts the regulatory activity of the domain, leading to accelerated protein turnover and almost completely reduced catalytic function. The third novel variant we found, the 4-bp deletion c.541_544delGAGG, causes a frameshift and generates a premature stop codon that leads to the absence of partial CD and whole MD, thus generating a truncated version of PAH (PAH Glu181Lysfs*13; 192 aa). Our results show that the homozygous deletion severely affects PAH protein level and enzyme activity (Figures 5B-D(Fig. 5)). In conclusion, according to molecular genetics, biochemical, and structural modeling results, the two novel deletions *PAH*: c.206_208delCTT and *PAH*: c.541_544delGAGG are severe pathogenic variations, significantly influencing the degradation, activation, tetramerization, and catalyzation processes of PAH proteins.

However, this study has several limitations that should be acknowledged. First, the sample size for each novel variant was relatively small, with only five patients across three variants, which limits the statistical power to establish robust genotype-phenotype correlations. Second, we did not perform mechanistic experiments to elucidate the molecular pathways leading to disease, such as investigations of mutant PAH protein degradation, tetramer formation stability, or BH_4_ responsiveness. Third, *in vivo* disease modeling using animal systems (e.g., mice or zebrafish) was not conducted to validate the pathogenicity of these variants in a physiological context. In our future studies, we plan to expand the PKU and HPA patient cohort to increase sample sizes for each variant category. Additionally, we will conduct comprehensive mechanistic studies, including but not limited to: (1) investigating how mutant PAH protein degradation occurs using pulse-chase assays and proteasome inhibition experiments; (2) assessing tetramer formation stability through native gel electrophoresis and size-exclusion chromatography; (3) evaluating BH_4_ responsiveness through *in vitro* enzyme assays with varying BH_4_ concentrations; and (4) developing *in vivo* disease models using mice or zebrafish to validate pathogenic mechanisms and test potential therapeutic interventions. These future studies will provide deeper insights into the molecular pathogenesis of PKU and may identify novel therapeutic targets for personalized medicine approaches*.*

In addition, the significant regional variability in HPA incidence, as highlighted by epidemiological studies, emphasizes the critical importance of adequate sample sizes in genetic studies. While our study identified three novel variants in the Chinese population, the relatively small number of patients per variant limits our ability to accurately estimate allele frequencies and establish robust genotype-phenotype correlations. Future multi-center collaborative studies with larger cohorts are essential to validate our findings and to comprehensively characterize the mutation spectrum in the Chinese PKU/HPA population.

## Conclusion

In summary, to further prevent the prevalence of HPA, we carried out a long-term and wide-ranging genetic screen among patients with HPA or PKU in Southern and Northern China. Through molecular analysis of five patients with PKU and their family members, we identified three novel variations, the 3-bp deletion c.206_208delCTT, the 4-bp deletion c.541_544delGAGG, and the missense variant c. 271C>A. We showed that the two deletions significantly affect PAH protein expression level and enzymatic activity. Taken together, genetic, biochemical, and structural modeling data, combined with clinical evidence, indicated that the two deletions were pathogenic and responsible for the molecular etiopathology of PKU. Our work further broadens the known variation spectrum of the human *PAH* gene and promotes our understanding of the molecular basis of PKU etiopathogenesis. This will aid in more comprehensive screening, prenatal diagnosis, and genetic counseling of the PKU disease, and contribute to further reduction of newborn defects.

## Notes

Fan Yang and Hua-Feng Li contributed equally as first author.

Fan Yang and Ying Yu (Medical Genetics & Antenatal Diagnosis Center, Hainan Branch, Shanghai Children’s Medical Center, School of Medicine, Shanghai Jiao Tong University, Sanya, China No. 339, Yingbin Road, Jiyang District, Sanya City, 572022, Hainan Province, China; E-mail: yuying2020@126.com) contributed equally as corresponding author.

## Declaration

### Ethic approval statement

The protocol of this study was reviewed and approved by the Ethics Committee of Hainan Branch, Shanghai Children's Medical Center, School of Medicine, Shanghai Jiao Tong University, Sanya (Approval No. SYFYIRB2023042), and the Ethics Committee of Women & Children's Health Care Hospital of Linyi (Approval No. 2013-YXLL-001). Written informed consent was obtained from all patients with PKU or HPA patients or their legal guardian, and their family members prior to enrollment. In addition, we have obtained the written informed consent of all participants to publish this data.

### Declaration of competing interest

The authors declare that they have no conflict of interest.

### Funding

This study was funded by the grants from the Basic Research Program of Guizhou Province (Grant No. QianKeHe Basics-ZK [2023] General 583), the Natural Science Foundation of Hainan Province (Grant No. 823RC617), the Major Research Project of “JinYe ZhongZi” (Grant No. JYZZ-ZD-202102), the Medicine and Health Science & Technology Project of Shandong Province (Grant No. 202301031323), and the Open Fund of Golden Coconut Seed - Key Laboratory of Molecular Medicine for Women and Children of Hainan Province (Grant No. 2025JYZZ-ZDSYS-06).

### Author contributions

Conceptualization, FY, HFL, and YY; data curation & formal analysis, FY, HFL, and WJT; funding acquisition, FY, HFL, and YY; methodology & software, FY, WJT, JPZ, JGQ, and TEC; writing of original draft, FY; review and editing, FY, HFL, WJT, JPZ, JGQ, TEC, LMY, and YY; resources: HFL, JPZ, JGQ, TEC, and YY; validation: FY, WJT, and JPZ; visualization: FY; project administration & supervision, FY, HFL, and YY.

### Artificial Intelligence (AI) - assisted technology

None was used in any stage of this work.

### Data availability statement

All data used and/or analyzed during this study may be available from the corresponding author on reasonable request.

### Acknowledgment

We sincerely thank all participants for participating in this study.

## Supplementary Material

Supplementary information

## Figures and Tables

**Table 1:**
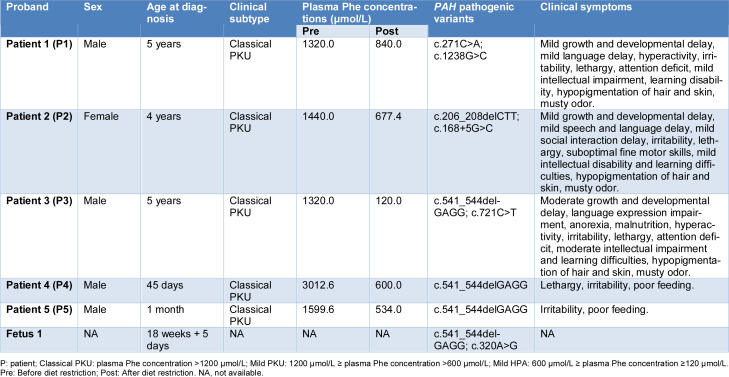
Clinical characteristics and biochemical data of five PKU patients carrying novel *PAH* variants

## References

[1] Acosta A, Silva W, Carvalho T, Gomes M, Zago M (2001). Mutations of the phenylalanine hydroxylase (PAH) gene in Brazilian patients with phenylketonuria. Human Mutation.

[2] Alibakhshi R, Moradi K, Mohebbi Z, Ghadiri K (2014). Mutation analysis of PAH gene in patients with PKU in western Iran and its association with polymorphisms: identification of four novel mutations. Metab Brain Dis.

[3] Anikster Y, Haack TB, Vilboux T, Pode-Shakked B, Thony B, Shen N (2017). Biallelic Mutations in DNAJC12 Cause Hyperphenylalaninemia, Dystonia, and Intellectual Disability. American Journal of Human Genetics.

[4] Bartholome K (1974). Letter: A new molecular defect in phenylketonuria. Lancet.

[5] Bickel H, Gerrard J, Hickmans EM (1953). Influence of phenylalanine intake on phenylketonuria. Lancet.

[6] Bjorgo E, Knappskog PM, Martinez A, Stevens RC, Flatmark T (1998). Partial characterization and three-dimensional-structural localization of eight mutations in exon 7 of the human phenylalanine hydroxylase gene associated with phenylketonuria. European Journal of Biochemistry.

[7] Blau N, van Spronsen FJ, Levy HL (2010). Phenylketonuria. Lancet.

[8] Bueno MA, Gonzalez-Lamuno D, Delgado-Pecellin C, Aldamiz-Echevarria L, Perez B, Desviat LR (2013). Molecular epidemiology and genotype-phenotype correlation in phenylketonuria patients from South Spain. Journal of Human Genetics.

[9] Buratti E, Chivers M, Kralovicova J, Romano M, Baralle M, Krainer AR (2007). Aberrant 5' splice sites in human disease genes: mutation pattern, nucleotide structure and comparison of computational tools that predict their utilization. Nucleic Acids Research.

[10] Cao YY, Qu YJ, Song F, Zhang T, Bai JL, Jin YW (2014). Fast clinical molecular diagnosis of hyperphenylalaninemia using next-generation sequencing-based on a custom AmpliSeq panel and Ion Torrent PGM sequencing. Molecular Genetics and Metabolism.

[11] Chen YF, Jia HT, Chen ZH, Song JP, Liang Y, Pei JJ (2015). Mutational spectrum of phenylketonuria in Jiangsu province. Eur J Pediatr.

[12] Chien YH, Chiang SC, Huang A, Chou SP, Tseng SS, Huang YT (2004). Mutation spectrum in Taiwanese patients with phenylalanine hydroxylase deficiency and a founder effect for the R241C mutation. Human Mutation.

[13] Couce ML, Boveda MD, Fernandez-Marmiesse A, Miras A, Perez B, Desviat LR (2013). Molecular epidemiology and BH4-responsiveness in patients with phenylalanine hydroxylase deficiency from Galicia region of Spain. Gene.

[14] Desviat LR, Perez B, Belanger-Quintana A, Castro M, Aguado C, Sanchez A (2004). Tetrahydrobiopterin responsiveness: results of the BH4 loading test in 31 Spanish PKU patients and correlation with their genotype. Molecular Genetics and Metabolism.

[15] Eiken HG, Knappskog PM, Apold J, Flatmark T (1996). PKU mutation G46S is associated with increased aggregation and degradation of the phenylalanine hydroxylase enzyme. Human Mutation.

[16] Erlandsen H, Fusetti F, Martinez A, Hough E, Flatmark T, Stevens RC (1997). Crystal structure of the catalytic domain of human phenylalanine hydroxylase reveals the structural basis for phenylketonuria. Nat Struct Biol.

[17] Fiori L, Fiege B, Riva E, Giovannini M (2005). Incidence of BH4-responsiveness in phenylalanine-hydroxylase-deficient Italian patients. Molecular Genetics and Metabolism.

[18] Fitzpatrick PF (2003). Mechanism of aromatic amino acid hydroxylation. Biochemistry.

[19] Fusetti F, Erlandsen H, Flatmark T, Stevens RC (1998). Structure of tetrameric human phenylalanine hydroxylase and its implications for phenylketonuria. The Journal of Biological Chemistry.

[20] Georgiou T, Ho G, Vogazianos M, Dionysiou M, Nicolaou A, Chappa G (2012). The spectrum of mutations identified in Cypriot patients with phenylalanine hydroxylase deficiency detected through neonatal screening. Clin Biochem.

[21] Gjetting T, Petersen M, Guldberg P, Guttler F (2001). In vitro expression of 34 naturally occurring mutant variants of phenylalanine hydroxylase: correlation with metabolic phenotypes and susceptibility toward protein aggregation. Molecular Genetics and Metabolism.

[22] Guldberg P, Mallmann R, Henriksen KF, Guttler F (1996). Phenylalanine hydroxylase deficiency in a population in Germany: mutational profile and nine novel mutations. Human Mutation.

[23] Guthrie R, Susi A (1963). A Simple Phenylalanine Method for Detecting Phenylketonuria in Large Populations of Newborn Infants. Pediatrics.

[24] Hillert A, Anikster Y, Belanger-Quintana A, Burlina A, Burton BK, Carducci C (2020). The Genetic Landscape and Epidemiology of Phenylketonuria. American Journal of Human Genetics.

[25] Hufton SE, Jennings IG, Cotton RG (1995). Structure and function of the aromatic amino acid hydroxylases. The Biochemical Journal.

[26] Jeannesson-Thivisol E, Feillet F, Chery C, Perrin P, Battaglia-Hsu SF, Herbeth B (2015). Genotype-phenotype associations in French patients with phenylketonuria and importance of genotype for full assessment of tetrahydrobiopterin responsiveness. Orphanet J Rare Dis.

[27] Kim SW, Jung J, Oh HJ, Kim J, Lee KS, Lee DH (2006). Structural and functional analyses of mutations of the human phenylalanine hydroxylase gene. Clinica Chimica Acta.

[28] Kobe B, Jennings IG, House CM, Michell BJ, Goodwill KE, Santarsiero BD (1999). Structural basis of autoregulation of phenylalanine hydroxylase. Nat Struct Biol.

[29] Kostandyan N, Britschgi C, Matevosyan A, Oganezova A, Davtyan A, Blau N (2011). The spectrum of phenylketonuria genotypes in the Armenian population: identification of three novel mutant PAH alleles. Molecular Genetics and Metabolism.

[30] Kure S, Hou DC, Ohura T, Iwamoto H, Suzuki S, Sugiyama N (1999). Tetrahydrobiopterin-responsive phenylalanine hydroxylase deficiency. J Pediatr.

[31] Lee DH, Koo SK, Lee KS, Yeon YJ, Oh HJ, Kim SW (2004). The molecular basis of phenylketonuria in Koreans. Journal of Human Genetics.

[32] Lee YW, Lee DH, Kim ND, Lee ST, Ahn JY, Choi TY (2008). Mutation analysis of PAH gene and characterization of a recurrent deletion mutation in Korean patients with phenylketonuria. Exp Mol Med.

[33] Leuzzi V, Carducci C, Carducci C, Chiarotti F, Artiola C, Giovanniello T (2006). The spectrum of phenylalanine variations under tetrahydrobiopterin load in subjects affected by phenylalanine hydroxylase deficiency. J Inherit Metab Dis.

[34] Liang Y, Huang MZ, Cheng CY, Chao HK, Fwu VT, Chiang SH (2014). The mutation spectrum of the phenylalanine hydroxylase (PAH) gene and associated haplotypes reveal ethnic heterogeneity in the Taiwanese population. Journal of Human Genetics.

[35] Lichter-Konecki U, Vockley J (2019). Phenylketonuria: Current Treatments and Future Developments. Drugs.

[36] MacDonald A, van Wegberg AMJ, Ahring K, Beblo S, Belanger-Quintana A, Burlina A (2020). PKU dietary handbook to accompany PKU guidelines. Orphanet J Rare Dis.

[37] Martinez A, Knappskog PM, Olafsdottir S, Doskeland AP, Eiken HG, Svebak RM (1995). Expression of recombinant human phenylalanine hydroxylase as fusion protein in Escherichia coli circumvents proteolytic degradation by host cell proteases. Isolation and characterization of the wild-type enzyme. The Biochemical Journal.

[38] Michiels L, Francois B, Raus J, Vandevyver C (1998). Identification of seven new mutations in the phenylalanine hydroxylase gene, associated with hyperphenylalaninemia in the Belgian population. Human Mutation.

[39] Mirisola MG, Cali F, Gloria A, Schinocca P, D'Amato M, Cassara G (2001). PAH gene mutations in the Sicilian population: association with minihaplotypes and expression analysis. Molecular Genetics and Metabolism.

[40] Murad H, Dabboul A, Moassas F, Alasmar D, Al-Achkar W (2013). Mutation spectrum of phenylketonuria in Syrian population: genotype-phenotype correlation. Gene.

[41] Okano Y, Asada M, Kang Y, Nishi Y, Hase Y, Oura T (1998). Molecular characterization of phenylketonuria in Japanese patients. Human Genetics.

[42] Okano Y, Hase Y, Kawajiri M, Nishi Y, Inui K, Sakai N (2004). In vivo studies of phenylalanine hydroxylase by phenylalanine breath test: diagnosis of tetrahydrobiopterin-responsive phenylalanine hydroxylase deficiency. Pediatr Res.

[43] Okano Y, Hase Y, Shintaku H, Araki K, Furuyama J, Oura T (1994). Molecular characterization of phenylketonuric mutations in Japanese by analysis of phenylalanine hydroxylase mRNA from lymphoblasts. Human Molecular Genetics.

[44] Okano Y, Kudo S, Nishi Y, Sakaguchi T, Aso K (2011). Molecular characterization of phenylketonuria and tetrahydrobiopterin-responsive phenylalanine hydroxylase deficiency in Japan. Journal of Human Genetics.

[45] Sarkissian CN, Shao Z, Blain F, Peevers R, Su H, Heft R (1999). A different approach to treatment of phenylketonuria: phenylalanine degradation with recombinant phenylalanine ammonia lyase. Proceedings of the National Academy of Sciences of the United States of America.

[46] Shi Z, Sellers J, Moult J (2012). Protein stability and in vivo concentration of missense mutations in phenylalanine hydroxylase. Proteins.

[47] Shintaku H, Kure S, Ohura T, Okano Y, Ohwada M, Sugiyama N (2004). Long-term treatment and diagnosis of tetrahydrobiopterin-responsive hyperphenylalaninemia with a mutant phenylalanine hydroxylase gene. Pediatr Res.

[48] Song F, Qu YJ, Zhang T, Jin YW, Wang H, Zheng XY (2005). Phenylketonuria mutations in Northern China. Molecular Genetics and Metabolism.

[49] Spaapen LJ, Bakker JA, Velter C, Loots W, Rubio-Gozalbo ME, Forget PP (2001). Tetrahydrobiopterin-responsive phenylalanine hydroxylase deficiency in Dutch neonates. J Inherit Metab Dis.

[50] Sterl E, Paul K, Paschke E, Zschocke J, Brunner-Krainz M, Windisch E (2013). Prevalence of tetrahydrobiopterine (BH4)-responsive alleles among Austrian patients with PAH deficiency: comprehensive results from molecular analysis in 147 patients. J Inherit Metab Dis.

[51] Su Y, Wang H, Rejiafu N, Wu B, Jiang H, Chen H (2019). The molecular epidemiology of hyperphenylalaninemia in Uygur population: incidence from newborn screening and mutational spectra. Ann Transl Med.

[52] Takarada Y, Kalanin J, Yamashita K, Ohtsuka N, Kagawa S, Matsuoka A (1993). Phenylketonuria mutant alleles in different populations: missense mutation in exon 7 of phenylalanine hydroxylase gene. Clinical Chemistry.

[53] Tao J, Li N, Jia H, Liu Z, Li X, Song J (2015). Correlation between genotype and the tetrahydrobiopterin-responsive phenotype in Chinese patients with phenylketonuria. Pediatr Res.

[54] Tao Y, Han D, Shen H, Li X (2021). Spectrum of PAH gene mutations and genotype-phenotype correlation in patients with phenylalanine hydroxylase deficiency from Shanxi province. Brain Dev.

[55] van Spronsen FJ, Blau N, Harding C, Burlina A, Longo N, Bosch AM (2021). Phenylketonuria. Nat Rev Dis Primers.

[56] Wang T, Okano Y, Eisensmith RC, Lo WH, Huang SZ, Zeng YT (1991). Identification of a novel phenylketonuria (PKU) mutation in the Chinese: further evidence for multiple origins of PKU in Asia. American Journal of Human Genetics.

[57] Waters PJ, Parniak MA, Akerman BR, Jones AO, Scriver CR (1999). Missense mutations in the phenylalanine hydroxylase gene (PAH) can cause accelerated proteolytic turnover of PAH enzyme: a mechanism underlying phenylketonuria. J Inherit Metab Dis.

[58] Waters PJ, Parniak MA, Akerman BR, Scriver C (2000). R. Characterization of phenylketonuria missense substitutions, distant from the phenylalanine hydroxylase active site, illustrates a paradigm for mechanism and potential modulation of phenotype. Molecular Genetics and Metabolism.

[59] Waters PJ, Parniak MA, Hewson AS, Scriver CR (1998). Alterations in protein aggregation and degradation due to mild and severe missense mutations (A104D, R157N) in the human phenylalanine hydroxylase gene (PAH). Human Mutation.

[60] Waters PJ, Parniak MA, Nowacki P, Scriver CR (1998). In vitro expression analysis of mutations in phenylalanine hydroxylase: linking genotype to phenotype and structure to function. Human Mutation.

[61] Xiang L, Tao J, Deng K, Li X, Li Q, Yuan X (2019). Phenylketonuria incidence in China between 2013 and 2017 based on data from the Chinese newborn screening information system: a descriptive study. BMJ Open.

[62] Zare-Karizi Sh, Hosseini-Mazinani SM, Khazaei-Koohpar Z, Seifati SM, Shahsavan-Behboodi B, Akbari MT (2011). Mutation spectrum of phenylketonuria in Iranian population. Molecular Genetics and Metabolism.

[63] Zhao S, Xiang J, Fan C, Asan, Shang X, Zhang X (2019). Pilot study of expanded carrier screening for 11 recessive diseases in China: results from 10,476 ethnically diverse couples. Eur J Hum Genet.

[64] Zhou YA, Ma YX, Zhang QB, Gao WH, Liu JP, Yang JP (2012). Mutations of the phenylalanine hydroxylase gene in patients with phenylketonuria in Shanxi, China. Genetics and Molecular Biology.

[65] Zhu T, Qin S, Ye J, Qiu W, Han L, Zhang Y (2010). Mutational spectrum of phenylketonuria in the Chinese Han population: a novel insight into the geographic distribution of the common mutations. Pediatr Res.

[66] Zhu T, Ye J, Han L, Qiu W, Zhang H, Liang L (2013). Variations in genotype-phenotype correlations in phenylalanine hydroxylase deficiency in Chinese Han population. Gene.

[67] Zurfluh MR, Zschocke J, Lindner M, Feillet F, Chery C, Burlina A (2008). Molecular genetics of tetrahydrobiopterin-responsive phenylalanine hydroxylase deficiency. Human Mutation.

[68] Zygulska M, Eigel A, Pietrzyk JJ, Horst J (1993). Phenylalanine hydroxylase gene: a novel splice mutation in intron 2 in two German and Polish families with severe phenylketonuria. Human Mutation.

