# Supplementary material for: Genetic diagnosis and molecular characterization of three novel variations in the phenylalanine hydroxylase gene from Chinese patients with phenylketonuria
Source: EXCLI J. 2026 Apr 10;25:458–75. doi: 10.17179/excli2026-9271 (PMC13139738; doi:10.17179/excli2026-9271)
Supplement: Supplementary information [file EXCLI-25-458-s-001.pdf]

EXCLI Journal 2026;25:458-475 – ISSN 1611-2156
Received: January 13, 2026, accepted: March 24, 2026, published: April 10, 2026

## Supplementary information to:

## Original article:

# GENETIC DIAGNOSIS AND MOLECULAR CHARACTERIZATION OF THREE NOVEL VARIATIONS IN THE *PHENYLALANINE HYDROXYLASE* GENE FROM CHINESE PATIENTS WITH PHENYLKETONURIA

Fan Yang[1,2,†,*], Hua-Feng Li[3,†],Wei-Jia Tang[4], Jin-Ping Zhu[3], Ji-Gang Qiu[3], Tian-E. Cai[5], Li-Mei Yu[6], Ying Yu[5,7*]

[1] Lishui Key Laboratory of Brain Health and Severe Brain Disorders. Lishui Second People's Hospital, Wenzhou Medical University, Lishui, China
[2] Bio-X Institutes, Key Laboratory for the Genetics of Developmental and Neuropsychiatric Disorders, Ministry of Education, Shanghai Jiao Tong University, Shanghai, China
[3] Department of Medical Genetics, Women & Children's Health Care Hospital of Linyi, Linyi, China
[4] The Research Center for Lin He Academician New Medicine, Institutes for Shanghai Pudong Decoding Life, Shanghai, China
[5] Medical Genetics & Antenatal Diagnosis Center, Hainan Branch, Shanghai Children's Medical Center, School of Medicine, Shanghai Jiao Tong University, Sanya, China
[6] Key Laboratory of Cell Engineering in Guizhou Province, Affiliated Hospital of Zunyi Medical University, Zunyi, China
[7] Key Laboratory of Molecular Medicine for Women and Children of Hainan Province, Hainan Branch, Shanghai Children's Medical Center, School of Medicine, Shanghai Jiao Tong University, Sanya, 572000, China

[†] These authors contributed equally to this work.

[*] **Corresponding author**: Ying Yu, Professor, Medical Genetics & Antenatal Diagnosis Center, Hainan Branch, Shanghai Children's Medical Center, School of Medicine, Shanghai Jiao Tong University, Sanya, China No. 339, Yingbin Road, Jiyang District, Sanya City, 572022, Hainan Province, China. E-mail: yuying2020@126.com

**Co-corresponding author**: Fan Yang, PhD, Associate Investigator, No. 69, Beihuan Road, Liandu District, Lishui Key Laboratory of Brain Health and Severe Brain Disorders, Lishui Second People's Hospital, Wenzhou Medical University, Lishui, 323000, Zhejiang Province, China. E-mail: yangfan@sibs.ac.cn

https://dx.doi.org/10.17179/excli2026-9271

This is an Open Access article distributed under the terms of the Creative Commons Attribution License (https://creativecommons.org/licenses/by/4.0/).

EXCLI Journal 2026;25:458-475 – ISSN 1611-2156
Received: January 13, 2026, accepted: March 24, 2026, published: April 10, 2026

**Supplementary Table S1: All of primers used in this study**

| Assay | Primer | Sequences (5' to 3') | Exon | Amplicon size (bp) |
|---|---|---|---|---|
| PCR & Sanger sequencing | *PAH*-E1F | GGAGATGCACCACGCAAGA | 1 | 413 |
| | *PAH*-E1R | GAAAGCCACCGAGGACAGA | | |
| | *PAH*-E2F | TGCCCTGGACTTACTTAT | 2 | 400 |
| | *PAH*-E2R | ATTCAAATCTGCCTGTTC | | |
| | *PAH*-E3F | GTTAGGTTTTCCTGTTCTGGTTCT | 3 | 443 |
| | *PAH*-E3R | AGTCTTCCAAGGCATTATTTCC | | |
| | *PAH*-E4F | GAGAATGTATTTGGGAATGGG | 4 | 363 |
| | *PAH*-E4R | AAGGTAAGAGGAAGGGAGGG | | |
| | *PAH*-E5F | TAACCAAGGGAAGGAGACAT | 5 | 210 |
| | *PAH*-E5R | CTCAACAAGCAAGGCAGAC | | |
| | *PAH*-E6F | TCCCTCTGCTAACCTAA | 6 | 369 |
| | *PAH*-E6R | ATACTTGCCTCCACATAC | | |
| | *PAH*-E7F | TGCCTAGCGTCAAAGCCTATGTCC | 7 | 349 |
| | *PAH*-E7R | AGCCAGCAATGAACCCAAACCTC | | |
| | *PAH*-E8F | TTTCCATTCTTTCTGCCC | 8 | 279 |
| | *PAH*-E8R | TACCTGGTTTCCGCTCTT | | |
| | *PAH*-E9F | AGGGTCTATGTGGGCTGTT | 9 | 461 |
| | *PAH*-E9R | GCCAAGGGTTTTCAAGG | | |
| | *PAH*-E10F | TCAGGTATCCCTTCATCCAGTCA | 10 | 350 |
| | *PAH*-E10R | CCCACAGCCATCATCAAATCA | | |
| | *PAH*-E11F | TGGGCTGTGATGTAGAAGGAA | 11 | 315 |
| | *PAH*-E11R | AGTGGCACCAGTCAGGAGG | | |
| | *PAH*-E12F | GGCTGTTGAAGACCCTGCTC | 12 | 320 |
| | *PAH*-E12R | ATGGCGATGGTAGGGAAAGA | | |
| | *PAH*-E13F | AGAAGCCCACTTATCCCCTA | 13 | 357 |
| | *PAH*-E13R | ATGACCCCAAAAGATTTACCA | | |
| qRT-PCR | *PAH* RT-F | CCTGACCCACATTGAATCTAG | 3 | 208 |
| | *PAH* RT-R | TCTGTCCAGCTCTTGAATGGT | 4 | |
| | *β-ACTIN* RT-F | GGCGGCACCACCATGTACCCT | 5 | 202 |
| | *β-ACTIN* RT-R | AGGGGCCGGACTCGTCATACT | 6 | |
| Site-directed mutagenesis | c.206_208del-F | AGACCTCG*TTTAAAGAAAGATGAGTA | NA | NA |
| | c.206_208del-R | CGAGGTCT*AGATTCAATGTGGGTC | NA | NA |
| | c.271C>A-F | GCTATGAC*AAACATCATCAAGATCTTG | NA | NA |
| | c.271C>A-R | GTCATAGC*AGGCAGGCTACGTTTATC | NA | NA |
| | c.541_544del-F | CATGAAGA*AAAGAAAACATGGGGCAC | NA | NA |
| | c.541_544del-R | TCTTCATG*TATTCCACTCGAGGGAT | NA | NA |

Asterisk denotes thiomodification. NA, not available.

**Supplementary Table S2: All variations detected in *PAH* gene of each subject from different family**

| Subject | Trivial name (protein effect) | Systematic name (DNA level) | Variation type | Genotype | Clinical significance |
|---|---|---|---|---|---|
| **Patient 1** | p.Arg413Pro | c.1238G>C | missense | heterozygous | pathogenic |
| | p.Leu91Met | c.271C>A | missense | heterozygous | likely benign |
| | p.Ser16Pro | c.46T>C | missense | heterozygous | uncertain significance |
| | p.Val245= | c.735G>A | synonymous | homozygous | benign |
| | IVS10+97 | c.1065+97G>A | silent | homozygous | benign |
| | p.Leu385= | c.1155C>G | synonymous | homozygous | benign |
| **Mother 1** | p.Arg413Pro | c.1238G>C | missense | heterozygous | pathogenic |
| | p.Leu91Met | c.271C>A | missense | heterozygous | likely benign |
| | p.Val245= | c.735G>A | synonymous | heterozygous | benign |
| | IVS10+97 | c.1065+97G>A | silent | heterozygous | benign |
| | p.Leu385= | c.1155C>G | synonymous | heterozygous | benign |
| **Father 1** | p.Leu91Met | c.271C>A | missense | heterozygous | likely benign |
| | p.Ser16Pro | c.46T>C | missense | heterozygous | uncertain significance |
| | IVS3-22 | c.353-22C>T | silent | homozygous | benign |
| | p.Val245= | c.735G>A | synonymous | homozygous | benign |
| | IVS10+97 | c.1065+97G>A | silent | homozygous | benign |
| | p.Leu385= | c.1155C>G | synonymous | homozygous | benign |
| **Patient 2** | IVS2+5 | c.168+5G>C | splicing | heterozygous | pathogenic |
| | p.Ser70del | c.206_208del | deletion | heterozygous | pathogenic |
| | IVS2+19 | c.168+19T>C | silent | heterozygous | benign |
| | p.Gln232= | c.696G>A | synonymous | homozygous | benign |
| | p.Leu385= | c.1155C>G | synonymous | homozygous | benign |
| **Mother 2** | IVS2+5 | c.168+5G>C | splicing | heterozygous | pathogenic |
| | 5’ UTR-71 | c.-71A>C | silent | heterozygous | benign |
| | IVS2+19 | c.168+19T>C | silent | heterozygous | benign |
| | IVS3-22 | c.353-22C>T | silent | heterozygous | benign |
| | p.Gln232= | c.696G>A | synonymous | heterozygous | benign |
| | p.Leu385= | c.1155C>G | synonymous | heterozygous | benign |
| **Father 2** | p.Ser70del | c.206_208del | deletion | heterozygous | pathogenic |
| | IVS3-22 | c.353-22C>T | silent | heterozygous | benign |
| | p.Gln232= | c.696G>A | synonymous | homozygous | benign |
| | p.Leu385= | c.1155C>G | synonymous | homozygous | benign |

EXCLI Journal 2026;25:458-475 – ISSN 1611-2156
Received: January 13, 2026, accepted: March 24, 2026, published: April 10, 2026

| Subject | Trivial name (protein effect) | Systematic name (DNA level) | Variation type | Genotype | Clinical significance |
|---|---|---|---|---|---|
| **Patient 3** | p.Glu181fs | c.541_544del | deletion | heterozygous | pathogenic |
| | p.Arg241Cys | c.721C>T | missense | heterozygous | pathogenic |
| | IVS3-22 | c.353-22C>T | silent | homozygous | benign |
| | p.Val245= | c.735G>A | synonymous | homozygous | benign |
| | p.Leu385= | c.1155C>G | synonymous | homozygous | benign |
| **Mother 3** | p.Arg241Cys | c.721C>T | missense | heterozygous | pathogenic |
| | IVS3-22 | c.353-22C>T | silent | homozygous | benign |
| | p.Val245= | c.735G>A | synonymous | homozygous | benign |
| | p.Leu385= | c.1155C>G | synonymous | homozygous | benign |
| **Father 3** | 5' UTR-71 | c.-71A>C | silent | heterozygous | benign |
| | IVS3-22 | c.353-22C>T | silent | heterozygous | benign |
| | p.Val245= | c.735G>A | synonymous | heterozygous | benign |
| | p.Leu385= | c.1155C>G | synonymous | heterozygous | benign |
| **Patient 4** | p.Glu181fs | c.541_544del | deletion | heterozygous | pathogenic |
| | IVS3-22 | c.353-22C>T | silent | homozygous | benign |
| | p.Val245= | c.735G>A | synonymous | homozygous | benign |
| | p.Leu385= | c.1155C>G | synonymous | homozygous | benign |
| **Mother 4** | p.Glu181fs | c.541_544del | deletion | heterozygous | pathogenic |
| | IVS3-22 | c.353-22C>T | silent | homozygous | benign |
| | p.Val245= | c.735G>A | synonymous | homozygous | benign |
| | p.Leu385= | c.1155C>G | synonymous | homozygous | benign |
| **Patient 5** | p.Glu181fs | c.541_544del | deletion | heterozygous | pathogenic |
| | IVS1+62 | c.60+62C>T | silent | heterozygous | benign |
| | IVS1+134 | c.60+134A>G | silent | heterozygous | benign |
| | IVS2+19 | c.168+19T>C | silent | heterozygous | benign |
| | IVS3-22 | c.353-22C>T | silent | homozygous | benign |
| | p.Val245= | c.735G>A | synonymous | homozygous | benign |
| | IVS10+97 | c.1065+97G>A | silent | homozygous | benign |
| | p.Leu385= | c.1155C>G | synonymous | homozygous | benign |
| **Mother 5** | p.Glu181fs | c.541_544del | deletion | heterozygous | pathogenic |
| | IVS1+62 | c.60+62C>T | silent | heterozygous | benign |
| | IVS1+134 | c.60+134A>G | silent | heterozygous | benign |
| | IVS3-22 | c.353-22C>T | silent | heterozygous | benign |
| | p.Val245= | c.735G>A | synonymous | heterozygous | benign |
| | IVS10+97 | c.1065+97G>A | silent | heterozygous | benign |
| | p.Leu385= | c.1155C>G | synonymous | heterozygous | benign |

EXCLI Journal 2026;25:458-475 – ISSN 1611-2156
Received: January 13, 2026, accepted: March 24, 2026, published: April 10, 2026

**Supplementary Table S3: *PAH* variants carried by subjects from the 110 families with PKU or HPA and the corresponding plasma phenylalanine concentrations**

| Proband | Sex | Age at diagnosis/ prenatal screening | Clinical subtype | Plasma Phe concentrations (µmol/L) | | *PAH* variants |
|---|---|---|---|---|---|---|
| | | | | Pre | Post | |
| **P1** | Male | 5 years | Classical PKU | 1320.0 | 840.0 | c.271C>A; c.1238G>C |
| **P2** | Female | 4 years | Classical PKU | 1440.0 | 677.4 | c.206_208delCTT; c.168+5G>C |
| **P3** | Male | 5 years | Classical PKU | 1320.0 | 120.0 | c.541_544delGAGG; c.721C>T |
| **P4** | Male | 45 days | Classical PKU | 3012.6 | 600.0 | c.541_544delGAGG |
| **P5** | Male | 1 month | Classical PKU | 1599.6 | 534.0 | c.541_544delGAGG |
| **P6 (F1)** | NA | Preg: 18 w +5 d | NA | NA | NA | c.541_544delGAGG; c.320A>G |
| **P7** | Male | 2 years | Classical PKU | 1368.0 | 54.0 | c.158G>A; c.308G>A; c.728G>A |
| **P8** | Male | 7 years | Classical PKU | 1260.0 | 336.0 | c.466G>C; c.728G>A |
| **P9** | Female | 2.5 years | Mild PKU | 1080.0 | 403.2 | c.728G>A; c.1162G>A |
| **P10** | Female | 3 years | Classical PKU | 1440.0 | 375.6 | c.442-1G>A; c.1197A>T |
| **P11** | Female | 1.6 years | Classical PKU | 2196.0 | 192.6 | c.1199G>C; c.1238G>C |
| **P12** | Female | 9 years | Mild PKU | 1200.0 | 900.0 | c.782G>A; c.1068C>A |
| **P13** | Female | 1 year | Mild PKU | 792.0 | 47.4 | c.442-1G>A; c.1252A>C |
| **P14** | Female | 5 years | Mild PKU | 1080.0 | 336.0 | c.721C>T; c.1068C>A |
| **P15** | Male | 7 years | Classical PKU | 1320.0 | 600.0 | c.208_210del; c.442-1G>A |
| **P16** | Female | 3 years | Classical PKU | 1440.0 | 90.0 | c.46T>C |
| **P17** | Female | 6 years | Classical PKU | 1500.0 | 561.0 | c.728G>A; c.46T>C |
| **P18** | Female | 4 years | Classical PKU | 1440.0 | 1042.2 | c.331C>T; c.699C>A |
| **P19** | Male | 7 years | Mild PKU | 960.0 | 539.4 | c.1223G>A; c.1301C>A |
| **P20** | Female | 3 years | Classical PKU | 1500.0 | 351.0 | c.728G>A; c.1068C>A |
| **P21** | Male | 5 years | Mild PKU | 960.0 | 386.4 | c.721C>T; c.728G>A |
| **P22** | Female | 7 years | Classical PKU | 1320.0 | 772.8 | c.611A>G; c.1068C>A |
| **P23** | Female | 4 years | Classical PKU | 1680.0 | 420.0 | c.331C>T; c.611A>G |
| **P24** | Male | 7 years | Mild PKU | 1080.0 | 659.4 | c.331C>T; c.611A>G |
| **P25** | Female | 2 years | Classical PKU | 2016.0 | 240.0 | c.442-1G>A; c.611A>G |
| **P26** | Female | 7 years | Mild HPA | 600.0 | 358.8 | c.721C>T; c.1197A>T |
| **P27** | Male | 3 years | Mild PKU | 780.0 | 354.0 | c.721C>T; c.1238G>C |
| **P28** | Female | 2 years | Mild HPA | 336.0 | 225.6 | c.320A>G; c.541_544delGAGG |
| **P29** | Male | 2 years | Mild PKU | 1098.0 | 202.2 | c.194T>C; c.1223G>A |
| **P30** | Female | 1 year | Classical PKU | 1286.4 | 375.6 | c.208_210del; c.331C>T |

EXCLI Journal 2026;25:458-475 – ISSN 1611-2156
Received: January 13, 2026, accepted: March 24, 2026, published: April 10, 2026

| | | | | | | |
|---|---|---|---|---|---|---|
| **P31** | Female | 1.5 years | Classical PKU | 2619.0 | 491.4 | c.442-1G>A; c.728G>A |
| **P32** | Female | 2.5 years | Classical PKU | 1500.0 | 397.2 | c.770G>T; c.755G>A |
| **P33** | Male | 8 months | Mild HPA | 382.2 | 94.2 | c.442-1G>A; c.721C>T |
| **P34** | Female | 4 years | Classical PKU | 1440.0 | 460.8 | c.728G>A; c.1197A>T |
| **P35** | Male | 3 years | Classical PKU | 1380.0 | 133.8 | c.721C>T; c.827T>G |
| **P36** | Male | 1 year | Classical PKU | 1496.4 | 584.4 | c.722del; c.728G>A |
| **P37** | Female | 2 years | Classical PKU | 1602.0 | 574.2 | c.728G>A; c.1197A>T |
| **P38** | Female | 1 year | Mild PKU | 834.0 | 270.0 | c.839A>G; c.1199G>C |
| **P39** | Female | 1 year | Mild HPA | 348.0 | 97.8 | c.442-1G>A; c.158G>A |
| **P40** | Male | 2 years | Mild PKU | 936.0 | 255.0 | c.331C>T; c.721C>T |
| **P41** | Male | 3 years | Classical PKU | 1500.0 | 380.4 | c.1045T>G; c.1197A>T |
| **P42** | Male | 3 years | Classical PKU | 1380.0 | 490.8 | c.1068C>A; c.1238G>C |
| **P43** | Male | 8 years | Mild PKU | 1080.0 | 943.2 | c.442-1G>A; c.1045T>G |
| **P44** | Male | 2 years | Mild HPA | 600.0 | 304.8 | c.158G>A; c.208_210del |
| **P45** | Male | 7 years | Mild PKU | 1200.0 | 615.6 | c.782G>A; c.1238G>C |
| **P46** | Male | 2 years | Mild HPA | 456.0 | 147.0 | c.721C>T; c.1197A>T |
| **P47** | Female | 8 years | Mild PKU | 1200.0 | 831.6 | c.977G>A; c.1197A>T |
| **P48** | Male | 2 years | Classical PKU | 1560.0 | 347.4 | c.331C>T; c.1139C>T; c.1199G>C |
| **P49** | Female | 11 years | Mild PKU | 900.0 | 525.0 | c.611A>G; c.1252A>C |
| **P50** | Female | 10 years | Classical PKU | 1320.0 | 601.2 | c.442-1G>A; c.331C>T |
| **P51** | Male | 4 years | Classical PKU | 1320.0 | 474.0 | c.611A>G; c.1238G>C |
| **P52** | Male | 9 years | Classical PKU | 1278.0 | 670.8 | c.611A>G; c.975C>G |
| **P53** | Male | 2 years | Mild HPA | 516.0 | 360.0 | c.168+5G>C; c.721C>T |
| **P54 (F2)** | NA | Preg: 18 w +6 d | NA | NA | NA | c.442-1G>A; c.1045T>G |
| **P55** | Female | 1.5 months | Mild PKU | 639.6 | 6 | c.728G>A; c.1197A>T |
| **P56 (F3)** | NA | Preg: 20 w +2 d | NA | NA | NA | c.331C>T; c.699C>A |
| **P57 (F4)** | NA | Preg: 18 w +1 d | NA | NA | NA | c.1223G>A; c.1301C>A |
| **P58 (F5)** | NA | Preg: 19 w | NA | NA | NA | c.1197A>T |
| **P59** | Female | 5 years | Classical PKU | 1380.0 | 760.8 | c.331C>T; c.1301C>A |
| **P60 (F6)** | NA | Preg: 18 w +6 d | NA | NA | NA | c.442-1G>A; c.1197A>T |
| **P61** | Female | 2 years | Classical PKU | 1653.0 | 217.2 | c.728G>A |
| **P62** | Female | 6 months | Classical PKU | 1939.8 | 118.8 | c.1238G>C; c.1301C>A |
| **P63** | Male | 1 year | Mild HPA | 244.2 | 156.0 | c.208_210del; c.301G>A |
| **P64** | Male | 2 months | Mild HPA | 325.8 | 78.0 | c.442-1G>A; c.158G>A |
| **P65 (F7)** | NA | Preg: 17 w +6 d | NA | NA | NA | c.331C>T; c.1139C>T; c.1199G>C |

| | | | | | | |
|---|---|---|---|---|---|---|
| **P66 (F8)** | NA | Preg: 19 w +5 d | NA | NA | NA | c.728G>A; c.1162G>A |
| **P67** | Male | 11 years | Classical PKU | 1501.8 | 283.2 | c.728G>A; c.1034_1041del |
| **P68** | Male | 2 months | Mild HPA | 495.6 | 298.2 | c.611A>G; c.728G>A |
| **P69** | Male | 4 years | Classical PKU | 1440.0 | 252.0 | c.194T>C; c.1197A>T |
| **P70** | Male | 5 years | Mild PKU | 660.0 | 271.2 | c.721C>T; c.728G>A |
| **P71** | Male | 2 months | Mild PKU | 1068.0 | 466.8 | c.740G>T; c.799C>G |
| **P72** | Male | 12 years | Classical PKU | 1500.0 | 493.8 | c.728G>A; c.1197A>T |
| **P73** | Female | 11 years | Classical PKU | 1320.0 | 900.0 | c.611A>G; c.728G>A |
| **P74** | Female | 6 years | Mild PKU | 1200.0 | 738.0 | c.977G>A; c.1197A>T |
| **P75** | Male | 1 year | Classical PKU | 2520.0 | 480.0 | c.1068C>A |
| **P76** | Male | 1.25 years | Mild PKU | 1140.0 | 360.0 | c.721C>T; c.775G>A |
| **P77** | Female | 3 months | Mild HPA | 417.6 | 245.4 | c.800A>T; c.1197A>T |
| **P78** | Male | 2 years | Mild PKU | 720.0 | 264.0 | c.721C>T; c.1238G>C |
| **P79** | Female | 15 years | Mild HPA | 690.0 | 480.0 | c.728G>A; c.1252A>C |
| **P80** | Male | 7 years | Classical PKU | 1500.0 | 270.0 | c.1045T>G; c.1197A>T |
| **P81** | Male | 8 years | Classical PKU | 1500.0 | 255.0 | c.977G>A; c.1197A>T |
| **P82** | Female | 8 years | Classical PKU | 1440.0 | 271.8 | c.250G>T; c.728G>A |
| **P83** | Male | 10 years | Classical PKU | 1320.0 | 720.0 | c.611A>G; c.975C>G |
| **P84** | Female | 2 months | Mild PKU | 900.0 | 7.8 | c.442-1G>A; c.728G>A |
| **P85** | Male | 2 months | Classical PKU | 2145.6 | 0.6 | c.722delG; c.1238G>C |
| **P86** | Female | 10 years | Mild PKU | 1200.0 | 960.0 | c.782G>A |
| **P87** | Female | 3 years | Classical PKU | 1500.0 | 1250.4 | c.728G>A; c.1199G>A |
| **P88** | Female | 4 years | Mild PKU | 757.8 | 402.0 | c.331C>T; c.770G>T |
| **P89** | Male | 3 months | Classical PKU | 2700.0 | 6.0 | c.611A>G; c.1068C>A |
| **P90** | Female | 4 years | Classical PKU | 2243.4 | 738.0 | c.728G>A; c.1197A>T |
| **P91** | Female | 12 years | Mild PKU | 760.2 | 630.0 | c.728G>A; c.1197A>T |
| **P92** | Female | 4 months | Mild PKU | 1080.0 | 760.8 | c.728G>A; c.740G>T |
| **P93** | Female | 1 year | Mild PKU | 720.0 | 480.0 | c.331C>T; c.1223G>A |
| **P94** | Male | 7 years | Classical PKU | 1920.0 | 288.6 | c.728G>A; c.1197A>T |
| **P95** | Male | 1 year | Mild HPA | 156.0 | 126.0 | c.158G>A; c.728G>A |
| **P96** | Female | 1 year | Classical PKU | 2418.0 | 307.8 | c.728G>A; c.977G>A |
| **P97** | Female | 12 years | Mild PKU | 1200.0 | 372.0 | c.331C>T; c.1238G>C |
| **P98** | Female | 7 years | Classical PKU | 1500.0 | 430.2 | c.722delG; c.755G>A |
| **P99** | Female | 6 years | Mild HPA | 498.0 | 283.8 | c.442-1G>A; c.1172G>C |
| **P100** | Male | 2.5 months | Mild PKU | 1098.0 | 81.6 | c.611A>G; c.728G>A |
| **P101** | Female | 2 months | Mild PKU | 720.0 | 318.0 | c.728G>A; c.1197A>T |
| **P102** | Male | 11 months | Classical PKU | 1974.0 | 69.6 | c.194T>C; c.728G>A |
| **P103** | Female | 2.5 months | Classical PKU | 1833.0 | 184.2 | c.775G>A; c.1197A>T |

EXCLI Journal 2026;25:458-475 – ISSN 1611-2156
Received: January 13, 2026, accepted: March 24, 2026, published: April 10, 2026

| | | | | | | |
|---|---|---|---|---|---|---|
| **P104** | Female | 2 years | Mild PKU | 720.0 | 264.0 | c.611A>G |
| **P105** | Female | 1 year | Classical PKU | 2580.0 | 173.4 | c.611A>G; c.1237C>A |
| **P106** | Female | 4 years | Classical PKU | 2341.2 | 259.8 | c.442-1G>A; c.707-1G>A |
| **P107** | Male | 6 years | Mild PKU | 1200.0 | 885.0 | c.728G>A; c.1065+39G>T |
| **P108** | Female | 2 years | Mild PKU | 1148.4 | 9.6 | c.728G>A; c.971T>A |
| **P109** | Male | 8 years | Classical PKU | 1290.0 | 408.0 | c.611A>G; c.1238G>C |
| **P110** | Female | 5 years | Classical PKU | 660.0 | 216.0 | c.721C>T; c.1238G>C |

P: patient; F: fetus; Preg: pregnancy; w: weeks; d: day(s). Classical PKU: plasma Phe concentration >1200 μmol/L; Mild PKU: 1200 μmol/L ≥ plasma Phe concentration >600 μmol/L; Mild HPA: 600 μmol/L ≥ plasma Phe concentration ≥120 μmol/L. Pre: Before diet restriction; Post: After diet restriction. NA, not available.
